# Case of Dropsy of the Ovum

**Published:** 1845-09

**Authors:** John E. H. Ligget

**Affiliations:** Frederick county, Maryland


### *Case of Dropsy of the Ovum.* By John E. H. Ligget, M. D., of Frederick county, Maryland.

On the 20th of July, 1843, I was desired to visit Mrs. D. H., (aged 41 years,) who gave the following history of her case. She is in the *sixth* month of her *ninth* pregnancy, but, with the exception of her general health being rather more delicate than usual under the circumstances, nothing remarkable was observed until within the last few weeks; when it was discovered that her abdomen was enlarging with unusual rapidity. She satisfied herself, however, by supposing the existence of twins, until the last fortnight preceding my visit, within which period the abdominal enlargement progressed to such an extent as to excite the most lively apprehensions in the minds of herself and friends. Upon examining the abdomen it was found more enlarged than is usual in twin cases, at the full period.

The swelling was tense and elastic, and a close examination did not enable me to detect the existence of fluctuation. Upon pressure over the abdominal parietes tenderness was not evinced, except in the right hypochondrium, where she states she has suffered from a dull, obtuse pain, for the last four or five years. Latterly this pain has been more acute, with tenderness on pressure. For some time her appetite has not been good, and the bowels have been confined. The renal secretion was of a deep reddish-brown colour, and not coagulable by heat; skin dry and harsh; pulse 110, sharp and tense; tongue covered with a short dirty white fur; thirst considerable; respiration easy, except when in a recumbent posture, when, of course, the abdominal pressure would create some embarrassment in that function. There was no infiltration into the subcutaneous cellular tissue. A review of the symptoms led me to suppose the existence of a collection of fluid in the peritoneal cavity, occasioned, probably, by obstructed circulation through the portal circle. A detail of the daily treatment would extend this communication to an inconvenient length. Suffice it to say, it consisted mainly in venesection, hydragogue cathartics, epispastics to the right hypochondrium,a nd a combination of mercurials with diuretics, in the following form.

℞. Hydrarg. chlorid. mit.
Digital. pulv., *aa.* gr. vj.
Potass. Nitrat. ʒj.
M. f. pulv. in chart. vj. divid.

One to be given every eight hours.

On the 24th the symptoms were unabated, and I was urged by my patient and her friends, to relieve her by the operation of *Paracentesis Abdominis.* This I declined for the present, and on the 25th, my brother, Doctor James Ligget, of Creagerstown, saw the case with me. He coincided in the views I had taken of the case, advised a continuance of the treatment, and agreed upon the impropriety of paracentesis, unless the respiration should become more embarrassed. The condition of my patient remained much the same, until the 1st of August, at which time the salivant effects of the mineral became obvious, when it was withdrawn and the Pulv. Scill. substituted.

The kidneys now took on increased action, and the urine assumed a more normal appearance. On the morning of the 3d August, I found Mrs. H. apparently much improved; the pulse was about 80, and soft; tongue cleaning; appetite improving, and the alvine evacuations natural. She was in fine spirits, and informed me she had passed about a quart of healthy looking urine, during the preceding night. After having been for some time engaged with her husband in an adjoining apartment, I was about to retire, when Mrs. H. informed me she had felt, within the last half hour, two or three slight pains, which she believed to be uterine. I of course remained, but as the pains had been very slight, did not examine *per vaginam.* Shortly after, whilst at the close stool, she stated she had "felt something give way," and immediately discharged about half a gallon of water. Now, for the first time, the *true* nature of the case flashed on my mind. I directed her to remain quiet, and left the room a moment to direct the arrangements that would be necessary for the complete evacuation of the uterus, which I

was aware must speedily follow. As soon as I left the room she imprudently got up and attempted to reach the close stool, but was prevented by a *rush of water*, which *poured from her in torrents.* Upon re-entering I found her standing on the floor supported by her husband, who had placed vessels to catch the water, about *three gallons of which had been discharged* during my temporary absence! She was immediately replaced on the bed, when, upon examining the vessels, I found in one of them a *well formed fœtus*, about 12 inches in length. By the rapidity of its expulsion the umbilical cord had been ruptured near its placental extremity. It lived about fifteen minutes. Upon returning to Mrs. H. the abdominal tumour was found to have very much subsided—the uterus could be felt pretty firmly contracted, though still larger than might be supposed to be occasioned by the mere retention of the secundines. A broad bandage was placed around her, and pinned moderately tight, and, after she had somewhat recovered from the exhaustion consequent upon the evacuation of so large a quantity of fluid, I proceeded to examine *per vaginam*, when I found the *head of a second fœtus presenting*, the membranes unruptured, and apparently containing very little fluid, The ruptured funis and placenta of the first fœtus could not be felt. At the end of an hour the pains recurred, irregularly, however, and without force, but attended with considerable hemorrhage. As the patient was already much exhausted, I deemed it hazardous longer to delay delivery. Accordingly I introduced my hand, ruptured the membranes, turned and delivered by the feet, a dead fœtus of smaller size than the former, upon which the hemorrhage ceased. After again allowing a sufficient time for repose, an effort was made, by the usual means, to excite the action of the uterus. In about forty-five minutes she felt some pain, and with it a return of the hemorrhage. Once more my hand was passed into the uterine cavity, for the purpose of removing its contents, and securing a permanent contraction. Upon passing up to the middle of the uterus my hand encountered a very firm "hour glass contraction," dividing the cavity into two chambers, the *inferior* of which was empty.

With infinite difficulty I dilated the strictured portion sufficiently to allow the passage of my fingers, but in attempting to press gently forward, it again contracted firmly upon my hand, causing a painful spasm of the muscles of the hand and arm. I could now feel the edge of a placenta, and upon passing my finger around it, discovered that it was *strongly adherent* to the walls of the uterus. Here was a multiplication of difficulties sufficient to have discouraged an older and more experienced accoucheur than myself; and to render my situation by no means an enviable one, I had been much indisposed all day with a severe attack of nervous headache, and had eaten nothing since the preceding evening. My hand in the uterus, a portion of which was firmly contracted upon it, cramping, paralyzing, and rendering it almost powerless ; the placenta adhering with unusual tenacity, and bidding defiance to my utmost efforts to detach it. After exerting myself until the perspiration rolled from me, and finding my strength failing, I withdrew my hand, told Mrs. H. of the difficulties by which the case was surrounded, and requested further professional aid. A messenger was immediately despatched for my able friend, Doct. Wm. Zollickoffer, and as the hemorrhage had again ceased, I sat down by the bed side to repose myself, desiring the patient to observe the most perfect quietude. A few moments afterwards, and a terrific *gush* from the uterus had well nigh destroyed my unfortunate patient. The countenance became pale and cadaverous—the pulse at the wrist imperceptible—an icy coldness crept over the extremities, and the heaving respiration gave evidence that the vital energies were nearly extinguished. Fortunately, however, there was but a gush, and the hemorrhage ceased. After waiting a few moments to see whether the system would rally, and finding the skin becoming damp and clammy, I directed *gin* (the only stimulant at hand) to be freely administered, with warm applications to the extremities. In half an hour I had the satisfaction to find my patient was rallying; shortly after which, at half past one o'clock, P. M., Dr. Zollikoffer arrived. After waiting some time, until Mrs. H. was sufficiently revived to bear the operation, we administered a portion of ergot, when Dr. Z., at my request, proceeded to extract the secundines. He found the "hour glass contraction" still existing, though probably less firm than previous to the last return of hemorrhage. Passing his hand into the superior chamber, after the lapse of considerable time, with great difficulty, and pausing frequently to rest himself, he finally succeeded in breaking up the adhesions which united one of the placentæ, *throughout its entire extent*, to the uterus. He now slowly and cautiously withdrew his hand, bringing with it the placentæ and membranes—a regular and firm contraction of the uterus being brought about, (as had been anticipated) by the action of the ergot. We remained with Mrs. H. until evening, when we left her doing well, although necessarily labouring under considerable prostration. From this period her convalescence was progressive. The appetite became good, the liver and kidneys resumed their functions, and tonics, and an occasional laxative, constituted the remainder of the treatment.

P. S. After the above was written, I was informed by Mr. H. that his wife was again pregnant, and that from the great abdominal enlargement, he feared the existence of another dropsical accumulation within the uterus. As her general health continued unimpaired, however, she did not come under treatment. On the 21st of April last I was summoned to visit her, and found that labor was actively progressing. In less than three hours after my arrival, she was delivered of two fine stout children, male and female, the head of the first and the feet of the second presenting as usual. In half an hour after the delivery of the second child hemorrhage supervened. This was found, on examination, to proceed, as in the former case, from an "hour glass contraction," and a consequent retention of the placenta, of which at this time there was but one. About one-third of the placenta was separated from the uterus—the balance adhered firmly, as upon the former occasion. With much difficulty I succeeded in removing it, and securing a natural contraction, after which my patient was lively and cheerful. There was no subsequent difficulty, except a slight attack of fever about three weeks after delivery, attended with a profuse leucorrhœal discharge, which yielded in three or four days to venesection, purgatives and diaphoretics. The mother and children are all at this time in the enjoyment of excellent health.

*July 30th*, 1845.

